# Cross-watershed distribution pattern challenging the elimination of *Oncomelania hupensis*, the intermediate host of *Schistosoma japonica*, in Sichuan province, China

**DOI:** 10.1186/s13071-022-05496-0

**Published:** 2022-10-11

**Authors:** Shen Chen, Ding Lu, Lei Duan, Ben Ma, Chao Lv, Yin-long Li, Shen-ning Lu, Lan-hua Li, Liang Xu, Zi-song Wu, Shang Xia, Jing Xu, Yang Liu, Shan Lv

**Affiliations:** 1 National Institute of Parasitic Diseases, China CDC (Chinese Center for Tropical Diseases Research); Key Laboratory on parasite and Vector Biology, National Health Commission; WHO Collaborating Centre for Tropical Diseases; National Center for International Research on Tropical Diseases, Ministry of Science and Technology, Shanghai, 200025 China; 2 Sichuan Center for Disease Control and Prevention, Chengdu, 610044 China; 3 Weifang Medical University, Weifang, 261053 China; 4 School of Global Health, Chinese Center for Tropical Diseases Research, Shanghai Jiao Tong University School of Medicine, Shanghai, 200025 China

**Keywords:** *Oncomelania hupensis*, Watershed, Population genetics, Spatial analysis

## Abstract

**Background:**

Snail control is critical to schistosomiasis control efforts in China. However, re-emergence of *Oncomelania hupensis* is challenging the achievements of schistosomiasis control. The present study aimed to test whether the amphibious snails can spread across watersheds using a combination of population genetics and geographic statistics.

**Methods:**

The digital maps and attributes of snail habitats were obtained from the national survey on *O. hupensis*. Snail sampling was performed in 45 counties of Sichuan Province. The *cox*1 gene of specimens was characterized by sequencing. Unique haplotypes were found for phylogenetic inference and mapped in a geographical information system (GIS). Barriers of gene flow were identified by Monmonier’s maximum difference algorithm. The watercourses and watersheds in the study area were determined based on a digital elevation model (DEM). Plain areas were defined by a threshold of slope. The slope of snail habitats was characterized and the nearest distance to watercourses was calculated using a GIS platform. Spatial dynamics of high-density distributions were observed by density analysis of snail habitats.

**Results:**

A total of 422 *cox*1 sequences of *O. hupensis* specimens from 45 sampling sites were obtained and collapsed into 128 unique haplotypes or 10 clades. Higher haplotype diversity in the north of the study area was observed. Four barriers to gene flow, leading to five sub-regions, were found across the study area. Four sub-regions ran across major watersheds, while high-density distributions were confined within watersheds. The result indicated that snails were able to disperse across low-density areas. A total of 63.48% habitats or 43.29% accumulated infested areas were distributed in the plain areas where the overall slope was < 0.94°. Approximately 90% of snail habitats were closer to smaller watercourses. Historically, high-density areas were mainly located in the plains, but now more were distributed in hilly region.

**Conclusions:**

Our study showed the cross-watershed distribution of *Oncomelania* snails at a large scale. Natural cross-watershed spread in plains and long-distance dispersal by humans and animals might be the main driver of the observed patterns. We recommend cross-watershed joint control strategies for snail and schistosomiasis control.

**Graphical Abstract:**

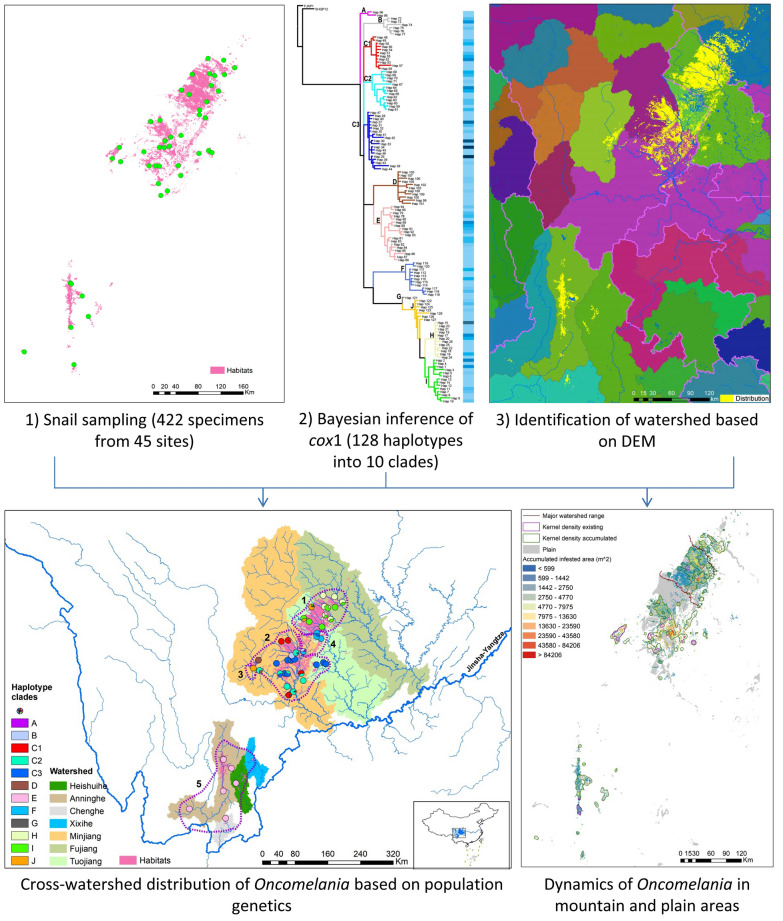

**Supplementary Information:**

The online version contains supplementary material available at 10.1186/s13071-022-05496-0.

## Background

Schistosomiasis japonica, a blood fluke infection, is endemic in China, Indonesia and the Philippines and has been eliminated in Japan [1, 2]. It was once a major infectious disease leading to 10 million human infections in China in the mid-1950s, but now is approaching elimination [3]. Among 450 endemic counties in China, > 96% have achieved transmission interruption or elimination of schistosomiasis [4]. Fewer than 10 cases were identified by stool examination from 11 million individuals across the country annually, although around 30,000 human cases with advanced schistosomiasis were recorded. A similar situation is observed in cattle, which is the most important reservoir of *Schistosoma japonicum* in China. The annual prevalence of schistosomiasis in cattle ranged from 0 to 51.9 per million between 2016 and 2020.

Snail control is an important component of schistosomiasis control in China. Snail control was once the essential prevention strategy before the advent of praziquantel, and thus, along with surveillance and treatment of humans and cattle, it is considered key in the national integrated strategy [5]. *Oncomelania hupensis* is the exclusive intermediate host species of *S. japonicum*. Its geographical distribution matches the endemicity of schistosomiasis well [6]. The overall prevalence of schistosomiasis across the country decreased markedly with the decline of areas infested by *O. hupensis* because of land transformation and water conservancy projects during the 1960s and 1970s [6]. Between the 1970s and 1990s, elimination of 98% of snail-infested areas and no snail discovery over 1 year were criteria for transmission control and transmission interruption respectively [7].

In response to the Sustainable Development Goals, schistosomiasis elimination is expected to be achieved in China by 2030 [8]. However, there are still many challenges ahead [8], two of which should be highlighted. The first is the re-emergence of *Oncomelania* snails in the areas where schistosomiasis was previously eliminated for several decades [6, 9, 10]. Historical evidence shows that the marked decrease of snail-infested areas not only facilitated the elimination of schistosomiasis but also benefitted maintenance of the status [11, 12]. Hence, a growing number of snail-infested areas could have an immediate impact on the endemicity of schistosomiasis, particularly in areas where the source of infections is not eliminated. The second challenge is natural infections in wild animals. Wild animals have been considered as an important source of infection for local foci in mountainous areas [13–15]. However, infections in wild animals are not included in the existing national surveillance system. The life cycle of *S. japonicum* can be maintained between wildlife and *O. hupensis* and is able to trigger the transmission and resurgence of schistosomiasis in humans.

Reasons for the re-emergence of *O. hupensis* were explored recently, including colonization of residual populations and re-introduction from other sources. The intensity of snail control and surveillance often decreased shortly after elimination of schistosomiasis [16, 17] and small residual populations of *O. hupensis* could expand to a certain extent before to be detected in routine surveillance [9, 18, 19]. Although 85% of the habitats of *O. hupensis* were eliminated in the whole country, the majority (84%) of eliminated habitats were still suitable for infestation [6]. Plant transportation and flooding are common reasons for re-introduction of *O. hupensis* in the eliminated habitats [20–23].

*O. hupensis* is an amphibious snail species. The adult snails normally live on the surface of wet soil with grass close to water bodies. Female adults lay eggs into soil where the eggs further develop. The eggs are brought into water by rain or flooding and then hatch. Young snails live in water and feed on microscopic organisms, e.g. algae. The ecological characteristics lead to a geographic distribution of *O. hupensis* along natural and manmade drainage. In addition, the velocity of water flow is also a crucial determinant of snail distribution. When the velocity of flow exceeds 2 m/s in the Yangtze River [24, 25] or 1 m/s in small channels in mountainous area [26], the adult snails cannot survive. Therefore, the habitats are often located in plain areas, particularly in the middle and lower reaches of Yangtze River [6].

Given *O. hupensis* is strictly distributed along drainage, watershed range should hinder the cross-watershed spread of *O. hupensis*. However, snails have been observed continuously distributed at the boundary of watersheds, such as the junction of Jiangxi Province (Yangtze River Basin) and Zhejiang Province (Qiantang River Basin) [6]. In this study, the distribution of *Oncomelania* in Sichuan province was investigated where both continuous distribution of *Oncomelania* at the intersections of different water systems (northern basin) and discrete distributions exist among watersheds (southern mountainous area). The study aimed to use spatial statistics and population genetics to characterise the distribution of *Oncomelania* snails across watersheds and to explore possible determinants.

## Methodology

### Data collection of *Oncomelania* habitats

The habitats of *Oncomelania* snails in the present study area were determined from a national survey on *O. hupensis* conducted in 2016 [6]. Briefly, the snail habitats were first identified by trained professionals in county-level institutes of schistosomiasis control according to the historical records since 1950s. The habitats were digitized by global positioning and geographic information systems. Surveys on *Oncomelania* snails were conducted in the field, categorizing the habitats into existing and historical groups. Habitat location and shape were obtained by digitalized maps if the habitats had been destroyed by land use change, e.g. urbanization.

### Population genetics of *Oncomelania* snails

*Oncomelania* snails from one habitat in each endemic county, if snails were endemic, were collected during the national survey in 2016. A systematic sampling method was used to collect snail specimens. Briefly, square sampling frames of 0.1 m × 0.1 m were placed at an interval of 5 m or 10 m depending on the length and width of the habitat. The snail density of the habitat was defined as the average number of living *Oncomelania* snails per frame. All living *Oncomelania* snails in the frames were collected. Snails were kept in 75% alcohol, and five to 20 snails from each site were randomly selected for DNA extraction. Snails were washed individually three times, and the soft body was taken out of the shell and immersed in pure water overnight. The tissues were cut into small pieces and incubated with sodium dodecylsulphate/proteinase K at 56 °C, pH 7.4, for 4–6 h. The suspension was centrifuged and the supernatant transferred into another tube for extraction. Snail DNA was extracted according using a DNeasy Blood & Tissue Kit (Qiagen, Germany). The DNA pellet was suspended in 30–50 μl H_2_O and kept at − 20 °C pending analysis.

The target gene of polymerase chain reaction (PCR) was *cox*1. The primers were designed according to a previous study [27], 5′-GGTCAACAAATCATAAAGATATTGG-3’ and 5′-TAAACTTCAGGGTGACCAAAAAATCA-3’. PCR was performed in 25 μl with 12.5 µl Taq Master Mix, 1 µl of each primer, 1.0 µl DNA sample and added water. The PCR cycling conditions used were 94 °C for 3 min and then 35 cycles at 94 °C for 60 s, 55 °C for 90 s and 72 °C for 60 s, followed by 72 °C for 7 min before final extension. The PCR products were visualized in a 1% agarose gel and recovered from the gel using mini-spin columns (Axygen). The purified products were used for sequencing using the dideoxynucleotide termination method.

The sequences determined in the present study were aligned using Clustal X version 2.0 [28] and trimmed as necessary using BioEdit version 7.0.9.0 (http://www.mbio.ncsu.edu/bioedit/bioedit.html) for subsequent analyses. The trimmed sequences were then analysed using DnaSP version 5.10.01 [29] to collapse into unique haplotypes. A network analysis was performed using TCS version 1.21 [30] to reveal the relation between haplotypes. The parsimony connection limit was set to 95% and separate networks were used to determine the clades.

To deepen our understanding of the phylogenetic placement of the haplotypes, a phylogenetic tree was constructed using Bayesian inference (BI), performed with MrBayes version 3.2.7 [31], using all haplotypes from the current study, including two haplotypes as outgroups from Shanghai and Fujian. Prior to BI, the best fit nucleotide substitution model (GTR + I + G) for this dataset was determined using a hierarchical likelihood ratio test in jModeltest version 2 [32]. The posterior probabilities were calculated using Markov chain Monte Carlo (MCMC) simulations. The average standard deviation of split frequencies was < 0.01, and the potential scale reduction factor was reasonably close to 1.0 for all parameters. A consensus tree was summarized and visualized using Mesquite version 3.70 [33].

Monmonier’s maximum difference algorithm [34] was used to assess sharp genetic discontinuities of *O. hupensis* populations in the study area, based on the Euclidean distance and coordinates of populations, using the Monmonier function of the adegenet package [35] for R [36]. In Monmonier, four runs were performed to find barriers that explain maximum genetic distances among populations.

Analysis of molecular variance (AMOVA) was performed in Arlequin version 3.5.2.2 [37] to explore the source of variation among the populations. Relation between genetic and geographical distances was examined using a Mantel test.

### River classification and watershed determination

Digital elevation model (DEM) data with a resolution of 90 m × 90 m for the study area were obtained from open source (https://srtm.csi.cgiar.org/). A watercourse, e.g. brook or stream, was determined by calculating flow direction and accumulation in ArcGIS 10.1 (ESRI, Redlands, CA, USA). D8 flow modeling was adopted by default for the flow analysis. The watercourses were extracted with a flow accumulation threshold. The results were evaluated by comparing to the watercourses produced in remote sensing image to determine the effectiveness of watercourses extraction. The watercourses were then classified into nine ranks using the Strahler method [38]. Briefly, the headwaters were considered as the first order and the downstream segments were defined at confluences (two streams running into each other). Watershed is the catchment area of watercourses. The division of watersheds is mainly based on the ridgeline between different watersheds. In this study, watersheds were determined in the study area by watershed analysis in ArcGIS 10.1. We determined the pour point (the point at which water flows out of an area) according to the river classification results and our own research needs. After the ridgelines and pour points had been determined, the study area was divided into watersheds.

### Plain characterization

We extracted all pixels of the DEM in selected snail habitats and plotted the frequency of each pixel value by slope. This distribution was notably right skewed. A slope value was considered as the threshold when the increase rate of frequency was stably < 1% backward (Additional file 1). We therefore defined a plain when the overall slope in the area was less than this threshold. The pixels with values less than threshold were extracted as potential plain areas. In the new raster layer the pixels beyond the plain areas were assigned no data. Some pixels with no data scattered in the target area affected the completeness of the plain. To mitigate the impact of pixels with no data, plain range was re-identified based on the analysis of point density in ArcGIS 10.1. We first transformed the pixels with values less than threshold slope to a point centralized in each pixel. We then generated a raster layer by calculating the point density with a radius of 10 pixels. The same pixel size as the original DEM was used for outputs. The pixels in the layer were categorized into two groups based on the density value by a method of natural breaks (Jeans). The group with higher values was extracted and transformed to polygon. We selected the snail habitats in the plain areas and calculated the total area infested by *O. hupensis*.

### Spatial analysis and statistics

The distance of each snail habitat to the nearest watercourse and its rank in a search radius of 5 km were calculated using the module of Analysis Tools in ArcGIS 10.1. We employed non-parametric test (Kruskal–Wallis) to compare the median distance for different watercourse ranks.

The geographical hotspots of snail distribution were determined by kernel density of habitats standardized by the accumulated infested area for the historical distribution and by the existing infested area for the present distribution. Briefly, the pixels in the raster layer of kernel density for historical distribution were categorized into two groups by natural breaks (Jeans). The two groups for present distribution were determined by the method of natural breaks mentioned above for historical distribution. The pixels with higher values were extracted and transformed to polygon as the range of hotspots.

## Results

### Population genetics

**Fig. 1:**
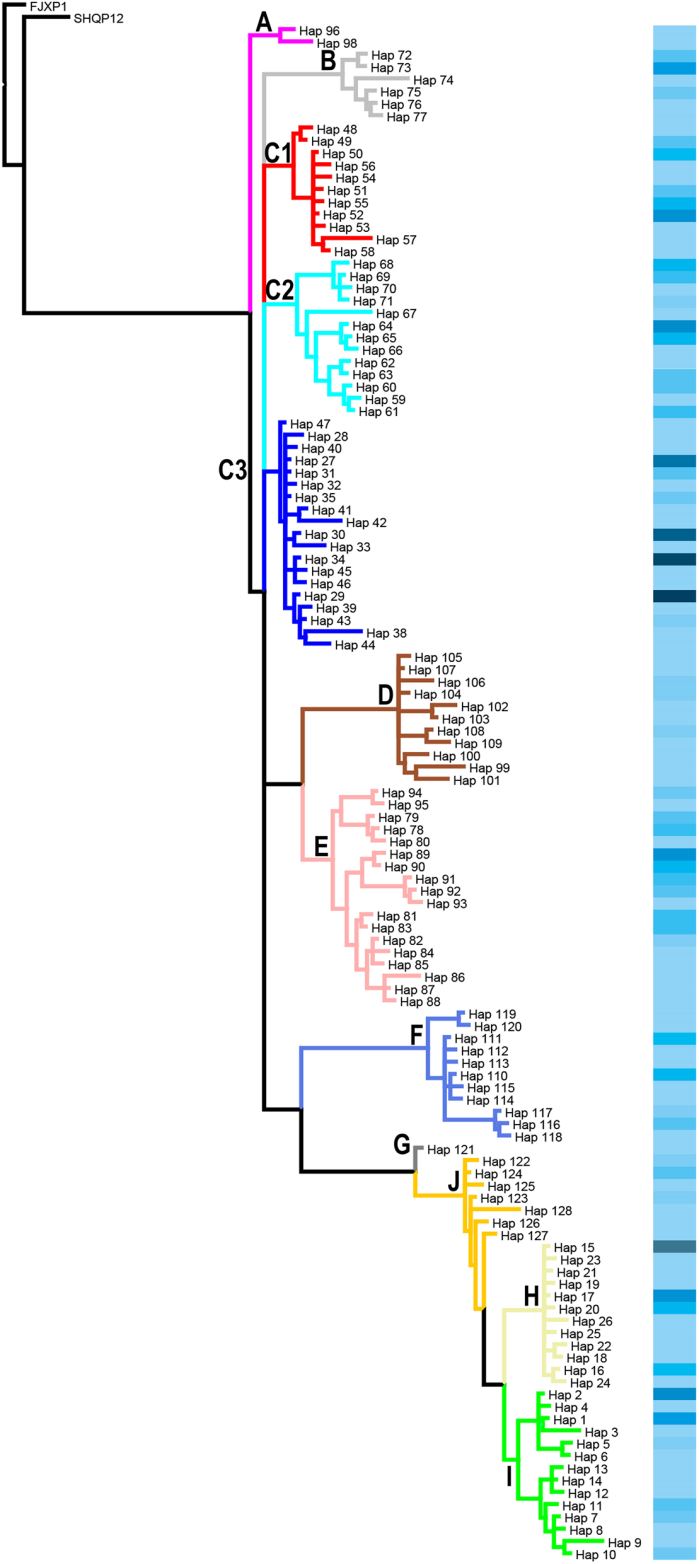
Phylogeny of 128 haplotypes derived from *cox*1 gene of *Oncomelania hupensis*. FJXP1 and SHQP12 are outgroups. Colors and bold letters on the branches denote different groups. The barcode on the right is the frequency of haplotypes

The *cox*1 gene of 422 snail samples from 45 collecting sites across the study area was characterized (Table 1). A total of 128 haplotypes were observed and the diversity was 0.9787 (Additional file 2). The network analysis identified 10 groups with a connection limit of 95%. The phylogeny of haplotypes based on Bayesian inference was consistent with the network analysis. To reveal the geographical pattern of the haplotypes, the large group C was further categorized into three subgroups named C1, C2 and C3 (Fig. 1). The three most common haplotypes were haplotype 29, 34 and 30, all of which were from Group C3.

**Table 1:** The clade composition and the number of snail individualsin sampling sites

ID	Long	Lat	County	Abbr	A	B	C1	C2	C3	D	E	F	G	H	I	J
1	104.417	31.377	Anzhou	AZ										9	1	
2	104.390	31.622	Beichuan	BC										16		
3	103.621	30.633	Chongzhou	CZ		1	13									
4	102.193	27.323	Dechang	DC							2					
5	103.545	29.907	Danlin	DL					12	2						
6	103.688	30.196	Dongpo	DP					12							
7	103.471	30.612	Dayi	DY			7									
8	103.595	29.621	Emeishan	EMS				4								
9	104.653	31.362	Fucheng	FC											4	
10	104.366	30.930	Guanghan	GH											7	
11	102.244	26.728	Huili	HL							10					
12	103.443	29.904	Hongya	HY				6	4							
13	103.609	29.899	Jiajiang	JJ				3	5							
14	104.640	31.593	Jiangyou	JY										14		
15	104.437	31.190	Jingyang	JYQ							1			1	1	
16	104.398	30.148	Jianyang	JYS					19							
17	103.952	29.750	Jinyan	JYX				5								
18	104.495	31.347	Luojiang	LJ										1	5	
19	104.345	30.672	Longquanyi	LQY								2				
20	102.967	30.199	Lushan	LS						10						
21	103.364	30.129	Mingshan	MS					4							
22	104.136	31.369	Mianzhu	MZ									1		1	6
23	102.474	27.497	Puge	PG							6					
24	103.609	30.198	Pujiang	PJ					7			1				
25	103.768	30.217	Pengshan	PS					17							
26	103.965	31.116	Pengzhou	PZ											8	5
27	104.255	30.797	Qingbaijiang	QBL								14				
28	103.699	30.302	Qionglai	QL		12										
29	103.912	29.908	Qingshen	QS			6	4								
30	104.244	30.101	Renshou	RS					15							
31	104.085	31.044	Shifang	SF											6	
32	103.956	30.392	Shuangliu	SL	2	5						7				
33	103.634	29.431	Shawan	SW			10		1							
34	102.856	30.021	Tianquan	TQ												1
35	103.739	29.504	Wutongqiao	WTQ			1	14			1					
36	102.186	28.032	Xicang	XC							14					
37	104.250	30.719	Xindu	XD					1			2				
38	102.201	28.018	Xide	XDB							5					
39	103.7918	30.3627	Xinjing	XJ				8	1							
40	101.448	26.932	Yanbian	YB							15					
41	102.990	29.971	Yucheng	YC				6		2						
42	104.452	30.114	Yanjiang	YJQ					16							
43	104.817	31.473	Youxian	YX										5	3	
44	104.555	31.099	Zhongjiang	ZJI										5	3	
45	102.375	27.824	Zhaojue	ZJU							4

Analysis of molecular variance (AMOVA) showed that 85.79% of variation was derived from inter-population differences (i.e. between sites). The estimated correlation coefficient between geographical distance (kilometre) and Fst was 0.42 (*P* < 0.01).

### Geographic barriers

**Fig. 2:**
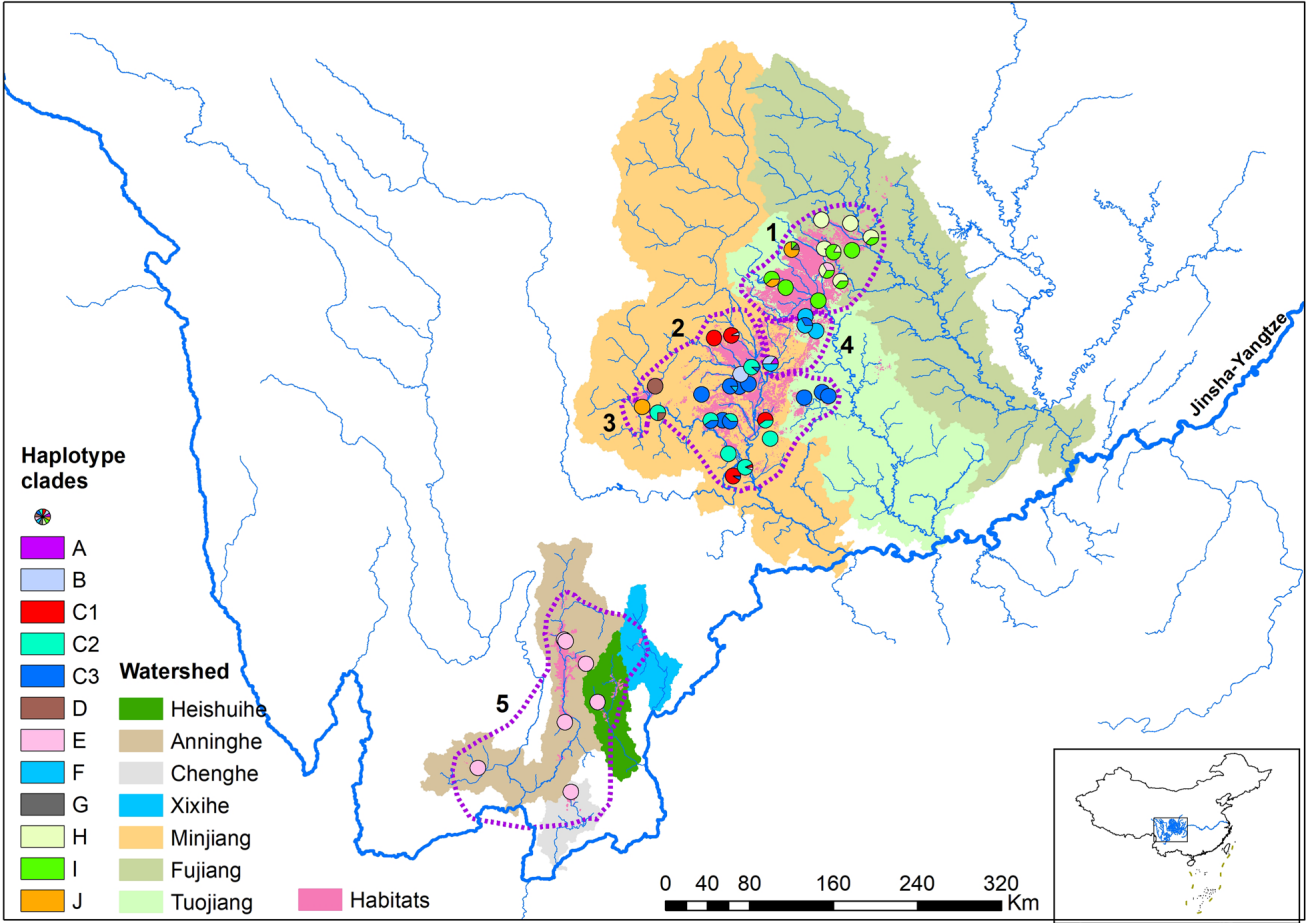
Geographical distribution of 12 clades in seven watersheds. Five sub-regions based on genetic barriers are denoted by dashed circles with Arabic numerals. The colors and letters for haplotype clades are consistent with Fig. 1

Snail habitats were distributed in seven watersheds directly connected to the Jinsha-Yangtze River. *O. hupensis* samples were collected from six out of the seven major watersheds, which were separated into two groups (Fig. 2). Three larger watersheds were located in the northeast and four smaller ones in the southwest. The haplotype diversity of the northeast group was higher than that of the southwest. Only clade E was observed in the southwest watersheds and it was genetically close to clade D, which was located in the west of watershed Minjiang.

Four potential genetic barriers for the snail populations were identified based on Monmonier’s algorithm (Additional file 3) and hence five subregions were drawn in the study area, shown in Fig. 2. All subregions except for subregion 3 ran across major watersheds. Meanwhile, four clades, i.e. H, I, C3 and F, were observed in two watersheds and one clade, E, was observed in five watersheds (Additional file 4). These findings indicated that haplotypes could spread through the range of watersheds.

### Distance to watercourses

**Fig. 3:**
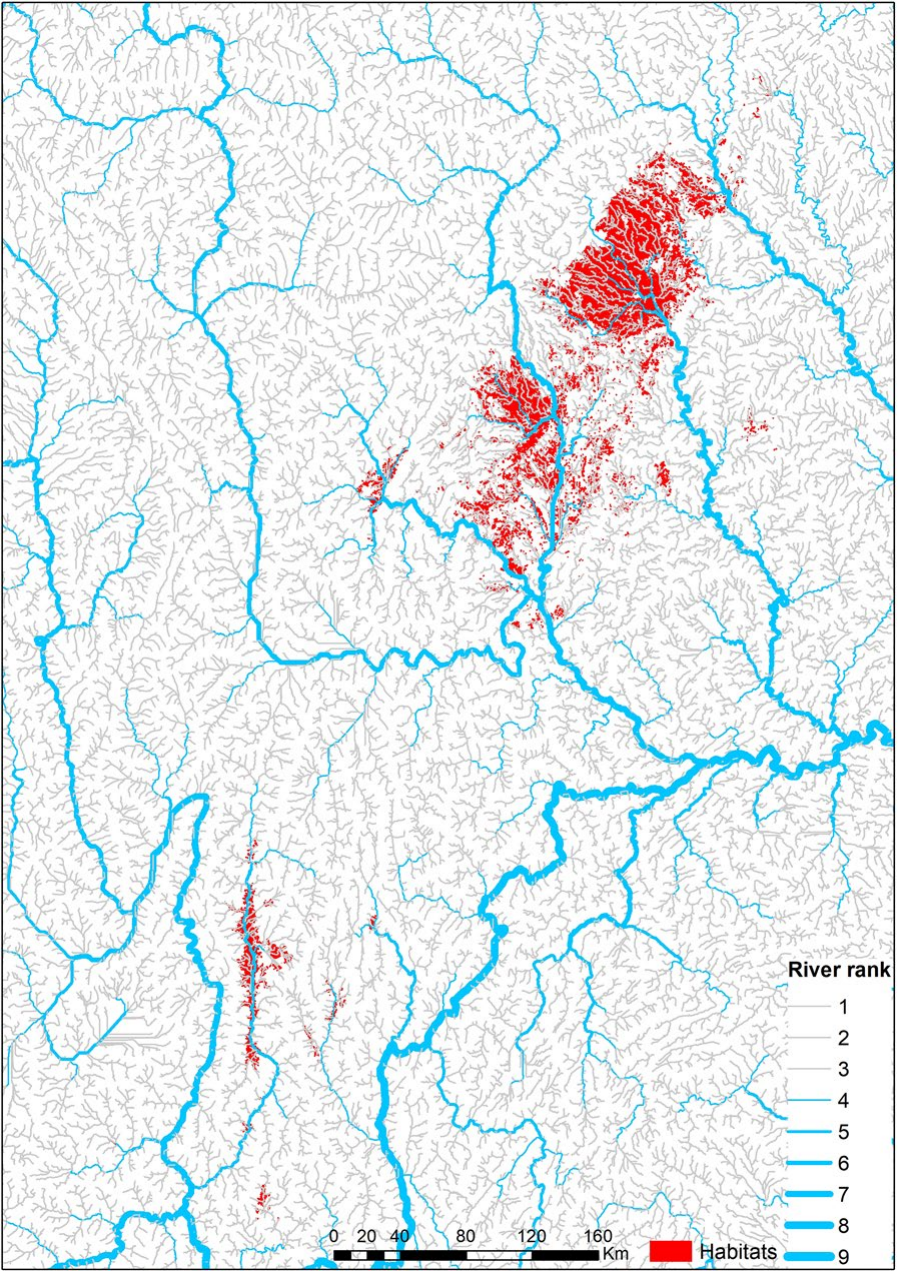
Relation between river rank and geographical distribution of snail habitats. Both historical and existing habitats of *Oncomelania hupensis* were included. The historical habitats, where *Oncomelania* snails do not infest any more, were digitalized and mapped based on the annual records since mid 1950s when the national schistosomiasis control programme commenced

**Fig. 4:**
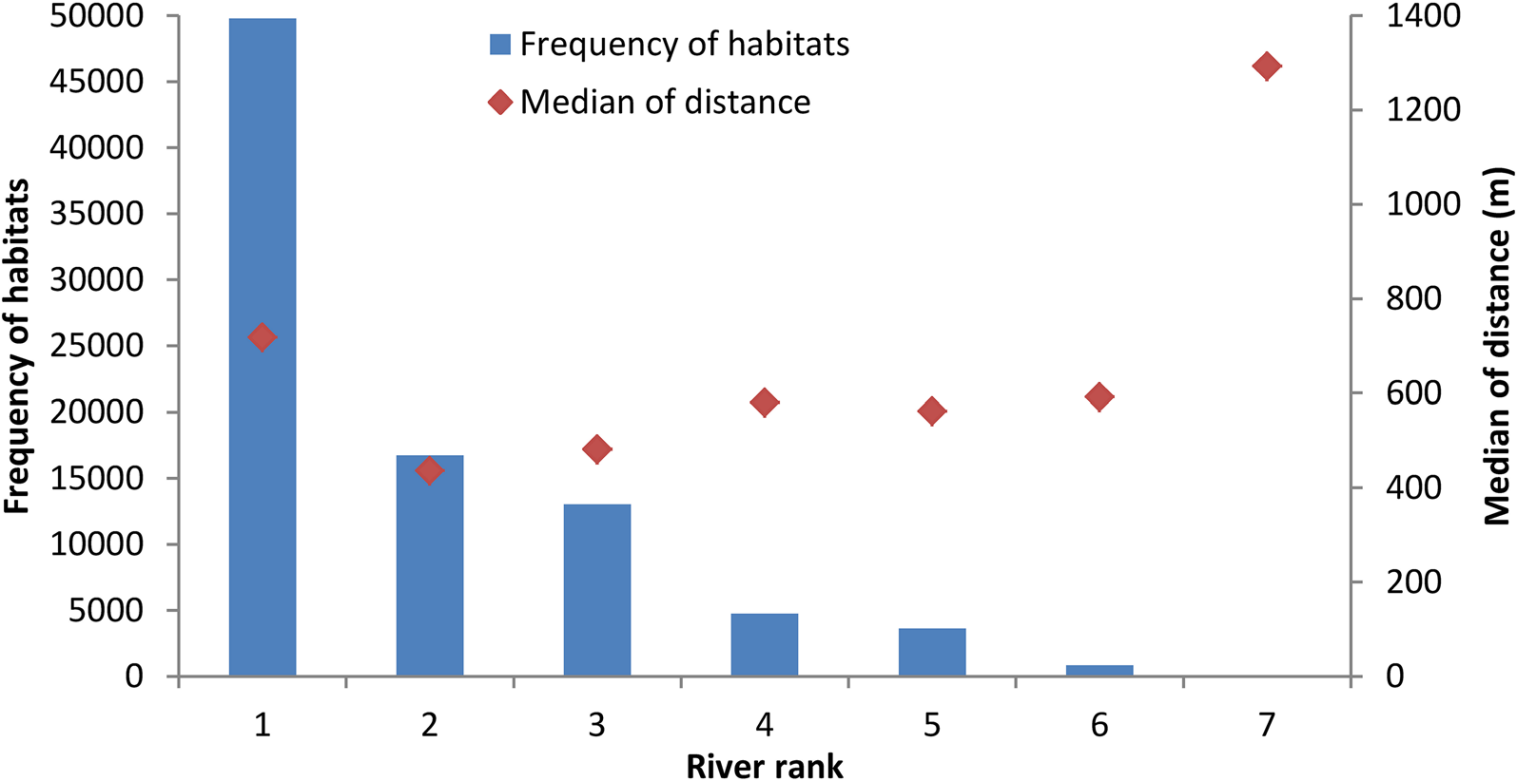
Frequency of snail habitats by shortest distance to either of seven river ranks

The watercourses (including streams and rivers) were categorized into nine ranks in the study areas according to the Strahler method. Approximately 90% of habitats are closer to low-ranked streams (rank 1 to 3; Fig. 3), of which the median distance was 598.35 m (Fig. 4). Habitats close to the streams of rank 1 alone accounted for 56.06% of all habitats with a median distance of 718.29 m. A Kruskal-Wallis (non-parametric) test indicated that the median distance between habitats close to rank 1 streams was significantly higher than for other habitat groups (Additional file 5).

### Plain area

**Fig. 5:**
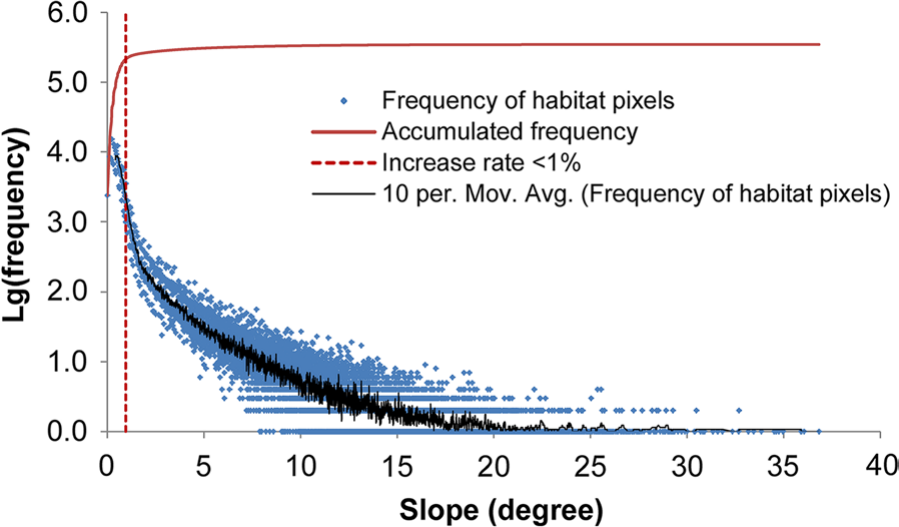
Frequency of snail habitat pixels by slope. The dashed line denotes the slope of 0.94°. The number of pixels more than the value sharply decreases

We calculated the slope of all pixels (90 m × 90 m) which were covered by snail habitats for a total of 346,026 pixels. About 95% of pixels have a slope < 8.99°. The frequency of pixels by slope was mapped as shown in Fig. 5. The increase rate (increment of pixel count per slope unit) sharply decreased at 0.94°. We therefore defined a threshold for *O. hupensis* distribution when the overall slope in the area was < 0.94°. All the pixels with a slope < 0.94° were extracted and transformed to a point feature dataset. Point density with a neighborhood radius of 10 pixels was then calculated. These new raster data were classified into two groups by a method of natural breaks (Jenks). The group with higher value was extracted as plain area.

**Fig. 6:**
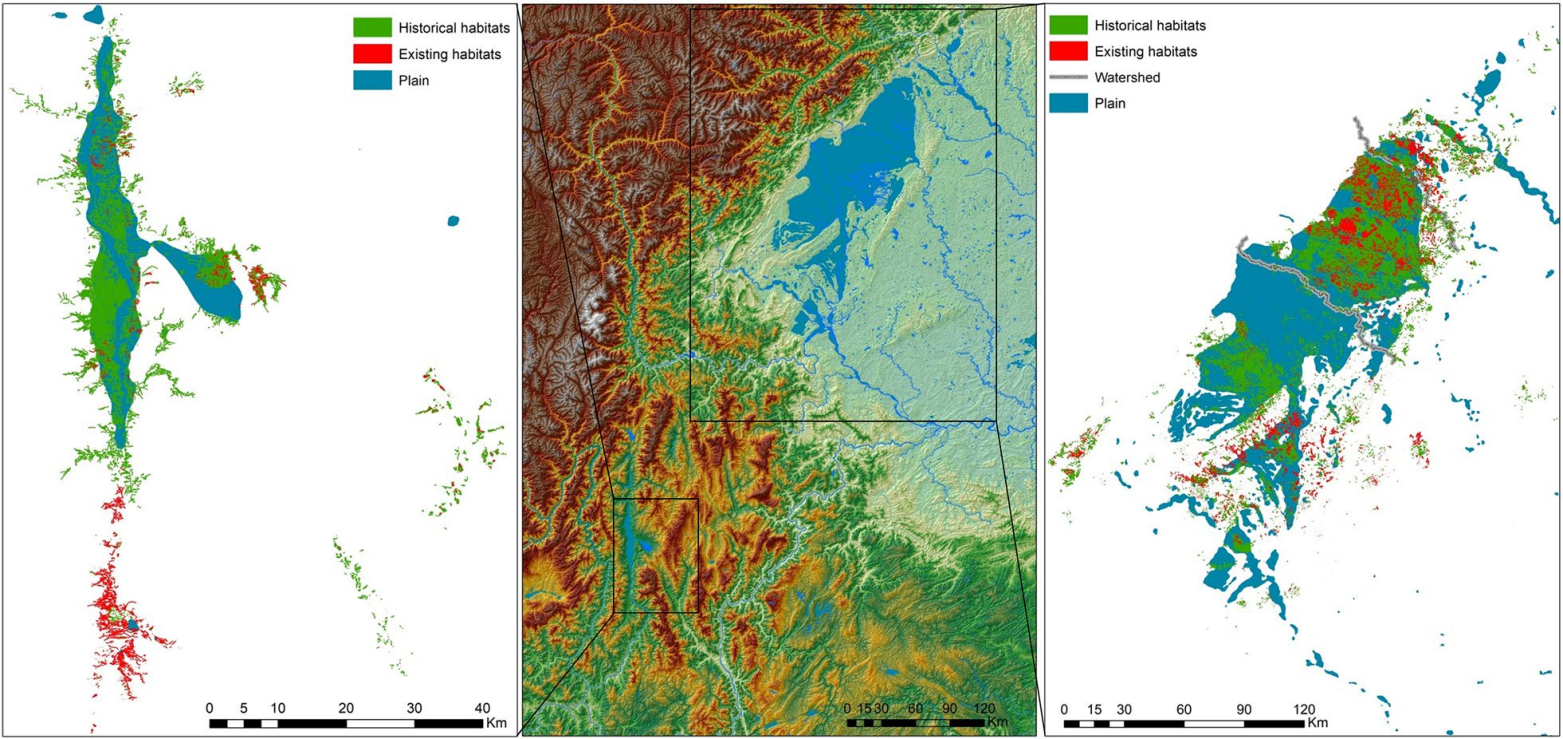
Snail habitats in major plains in study area. The middle sector shows the topography of study area and the plain areas with slope less than 0.94°. The right and left sectors denote the two major plains (right, larger and left, smaller)

A total of 53,714 habitats including infested areas of 127.92 million m^2^ were identified in plains, accounting for 60.48% and 43.29% of all habitats respectively (Table 2). Two major plains were identified in the present study (Fig. 6, middle). The large plain was at the northeast edge of Sichuan Basin (Fig. 6, right), where 50.21% of habitats were distributed. The small one was surrounded by the mountains located in southwest of the study area (Fig. 6, left), where 6.54% of habitats were distributed.

**Table 2:** The distribution of habitats in plain and mountain areas

	In plain		Outside plain	
	Number	Infested area (m^2^)	Number	Infested area (m^2^)
Existing habitats	10,953	13,881,507.00	8494	32,320,959.97
Eliminated habitats	42,761	114,034,937.10	26,603	139,206,300.90
Total	53,714	127,916,444.10	35,097	171,527,260.87

There were 10,953 existing habitats with an overall infested area of 13 million m^2^ in the plain areas. The number and the infested area of snail habitats in the plains have decreased by 79.61% and 89.15% in the last 6 decades respectively (Table 2). They were significantly higher than the decreases rate (75.80% and 81.16%) of snail habitats outside the plains according to the Chi-square test (*P* < 0.01).

**Fig. 7:**
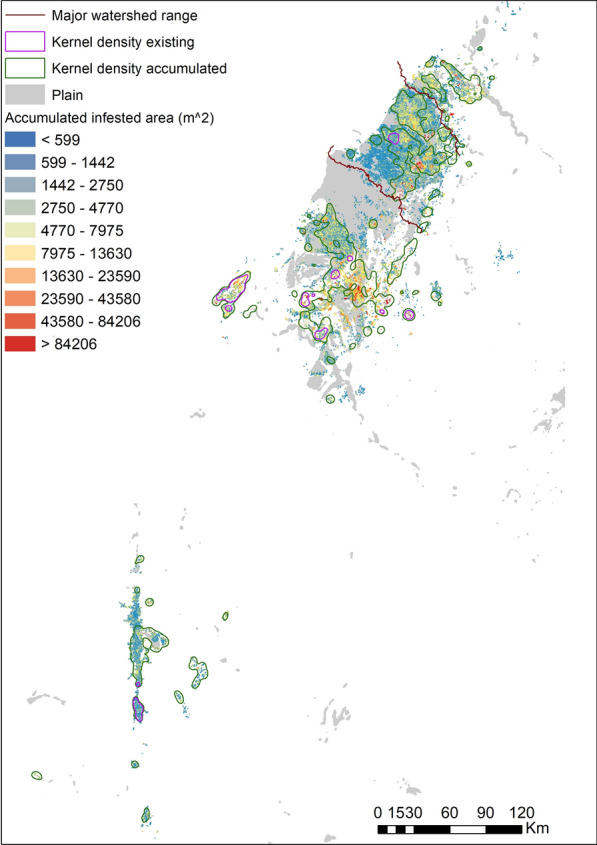
Change in high-density distributions of *O. hupensis* since the 1950s

The density of snail habitats, which was weighted by accumulated infested area, was characterized across the study area in ArcGIS. Many higher-density areas were distributed in the plains, particularly in the north part of the study region. However, the higher-density area of existing habitats, which indicated the current situation of snail distribution, had been observed shrinking and mainly located in the mountainous areas now (Fig. 7).

## Discussion

Although the study area (i.e. Sichuan province) is conventionally considered as a hilly or mountainous endemic area, one of three types of endemic area with schistosomiasis in China [39], *O. hupensis* was mainly distributed in the plains areas in the present study. This distribution characteristic is consistent with that in the middle and lower reaches of the Yangtze River, where most *Oncomlania* snails breed [6]. We also noted that some habitats occurred in the high-slope region in our study region. However, the field surveys showed that they were normally located in the flat ditches in manmade terrace land (e.g. paddy field) [40]. Therefore, habitats infested by *O. hupensis* were of low slope in either mountainous or plain areas, which may be a crucial factor to define the distribution range of *O. hupensis* in the country [41, 42].

We observed that most snail habitats were close to small watercourses. For example, > 56% snail habitats were nearest to water courses of rank 1. Furthermore, the percentage of habitats declined quickly by the rank of watercourses (shown in the Fig. 4). Interestingly, the median of nearest distance of habitats that were close to watercourses of rank 1 was significantly higher than that of other habitats that were close to higher ranked watercourses. The phenomenon implied that *O. hupensis* was likely to distribute in the smaller watercourses that were not identified and included in the present study because of the DEM resolution. Indeed, > 70% habitats in Sichuan province were small ditches [40]. Furthermore, few snail habitats were observed in the riverbed of high-rank watercourses or big rivers in the present study located in the upper stream of the Yangtze River. This was much different from the middle and lower streams where many snail habitats were distributed in the marshlands in lake and river beds [43, 44]. This may be explained by the slope and structure (rock) of riverbeds as well as runoff.

The national survey on *O. hupensis* showed that snail habitats in irrigation areas in the middle and lower reaches of the Yangtze River were eliminated quickly following the construction or repair during the 1960s and 1970s of flood control levees along the river and culverts at the channel entrances[6]. The disturbed hydrology of channels connecting irrigation areas with the Yangtze River, and the interrupted supply of snails from marshland might play a crucial role in elimination of snail habitats [45, 46]. In addition, the land use change in the plain area (urbanization, traffic construction, etc.) and the fragmentation of habitats accelerated the elimination of snails [47]. Our results showed that the high-density areas have shrunk considerably and are now mainly located in mountainous areas. The plains in the study area are the most economically developed and land use changes are also affecting the distribution of *Oncomelania* [40]. In addition, Sichuan Province has made great efforts to facilitate agricultural transformation. Many paddy fields where snails breed have been changed to dry lands where fruit trees are planted, which thus reduces the number and area of suitable snail habitats [48]. Although the number and area of snail habitats in both plain and mountainous areas were generally reduced in Sichuan Province, the proportion of eliminated habitats in mountainous areas was significantly lower. The snails were scattered in high land terraces, making it difficult to control and eliminate them by routine approaches, e.g. molluscicide. In addition, wild animals contributed to the maintenance of the natural life cycle of *S. japonicum* in hilly endemic areas [13–15]. Therefore, the focus of schistosomiasis control in the study area should be switched to the mountainous areas, along with necessary surveillance measures[49].

Several high-density areas or hotspots were identified in this study. The clustering distribution of haplotypes supported the relatively independent high-density areas. Although the hotspots were located within a few major watersheds, the cross-watershed spread of *Oncomelania* snails could be inferred from the population genetics analysis. Four out of five subregions derived from genetic barrier analysis run across major watersheds. In the traditional understanding, the distribution of amphibious *O. hupensis* is strictly in accordance with the water drainage system, and it is difficult to break through water system boundaries [26]. The cross-watershed spread of *O. hupensis* leads to the continuous importation of *O. hupensis* into new snail habitats, which makes it difficult to achieve a breakthrough in the control of *O. hupensis*. Two reasons may explain our results. First is that the ranges of watersheds were not able to form a geographic barrier to hinder the spread of snails. Although three adjacent watersheds were identified in the north of study area, a large plain ran across them and thus affected no geographic barriers on the boundaries. Second is the impact of human activities. Given *Oncomelania* snails commonly are distributed in irrigation ditches, the irrigation systems across watersheds in the plains can promote the spread of snails. Long distance dispersal by humans may also facilitate the cross-watershed spread, as indicated by the evidence of the same haplotypes (e.g. clade C3 and F) in separate populations in different watersheds. The extreme case for the effect of human activities is clade E. The haplotypes from clade E were mainly distributed in three watersheds in the south part of the study area, which are separated by high-elevation barriers. In addition, the same haplotype to the far southwest collecting site (Yanbian County) was observed in two counties in the north of the study area, although there was only one sample from each population. Our findings indicate that cross-watershed strategy for schistosomiasis control should be paid more attention, such as through construction of water conservancy facilities to limit *O. hupensis* migration across watershed and quarantine of plant imported from watershed infested by *O. hupensis*.

Our analysis showed that the distribution of *O. hupensis* could be basically divided into northern and southern groups. The haplotype diversity of the northern group was strikingly higher than that of the southern group. Clade E was the only clade in the southern group. The phylogeny indicated that the clades E and D originated from the same ancestor, which differentiated from the northern subgroups. Therefore, we inferred that the ancestor of clade E was accidentally introduced from the northern of study area and established its own differentiated populations. The time of introduction remains to be explored in future studies.

There are some limitations of our study. First, the slope threshold for plain definition was determined based on geographical statistics of spatial characteristics of our snail habitats. This might be different from the reality. Water flow velocity was considered an essential factor for the distribution of *O. hupensis* [26]. The slope of habitats could be determined based on water flow velocity by a hydrodynamic model, e.g. Chézy formula. However, no conclusive evidence was available to determine the threshold of water flow velocity that allows snails to remain and breed in the field [24, 25, 50, 51]. Further studies should be conducted to obtain the threshold of water flow velocity. Second, we did not use higher-resolution DEM in the present study and hence the slope value for many habitats was higher than the real situation. As a result, some habitats might be incorrectly assigned to hilly regions. Third, snail sampling size was small in some collecting sites because of very few living snails observed in the 2016 cross-sectional national survey and furthermore no more subsequent sampling since the survey. Meanwhile, it was also possible that some snail-infested areas had not been detected. In the present study, we included as many collecting sites as possible to overcome this.

## Conclusion

Our findings showed cross-watershed spread of *Oncomelania* snails at a large scale, which implied natural cross-watershed spread in plain areas and long-distance dispersal by humans and animals. Our research also suggested that the distribution of snails tends to be smaller-rank watercourses. Therefore, for the purposes of control, it is necessary to understand more completely the relationship between snail distribution and water systems at different geographic scales and in different ecological contexts. In practice, we recommend synchronized cross-watershed control strategies to maximise effectiveness and enhance the sustainability of schistosomiasis elimination in China.

## Supplementary Information

**Additional file 1**: **Figure S1**. The increase rate of frequency by slope from low to high. The increase rate of frequency was defined as the frequency at a specific slope was divided by the accumulated frequency at all observed slopes that were less than the specific slope.

**Additional file 2**: **Dataset S1**. The 130 unique *cox1* haplotypes of *Oncomelania* *hupensis* from this study including two outgroups

**Additional file 3**: **Figure S2**. Genetic barriers for the snail populations based on Monmonier’s algorithm.

**Additional file 4**: **Figure S3**. Three-dimensional distribution pattern of clades in populations, watersheds and frequency.

**Additional file 5**: **Figure S4**. A Kruskal-Wallis comparison analysis of median distances among different groups.

## Data Availability

All data or information used in the article, except for the spatial information of snail habitats, are provided in text or supplementary files. Since the survey of *Oncomelania* snail was conduct by Ministry of Health of China, the dataset of spatial information of snails used in analysis is not open. However, the data can be shared based on personal communication to corresponding author (lvshan@nipd.chinacdc.cn).
