# Supplementary figures and images for: Cross-watershed distribution pattern challenging the elimination of Oncomelania hupensis, the intermediate host of Schistosoma japonica, in Sichuan province, China
Source: Parasit Vectors. 2022 Oct 11;15:363. doi: 10.1186/s13071-022-05496-0 (PMC9555091; doi:10.1186/s13071-022-05496-0)

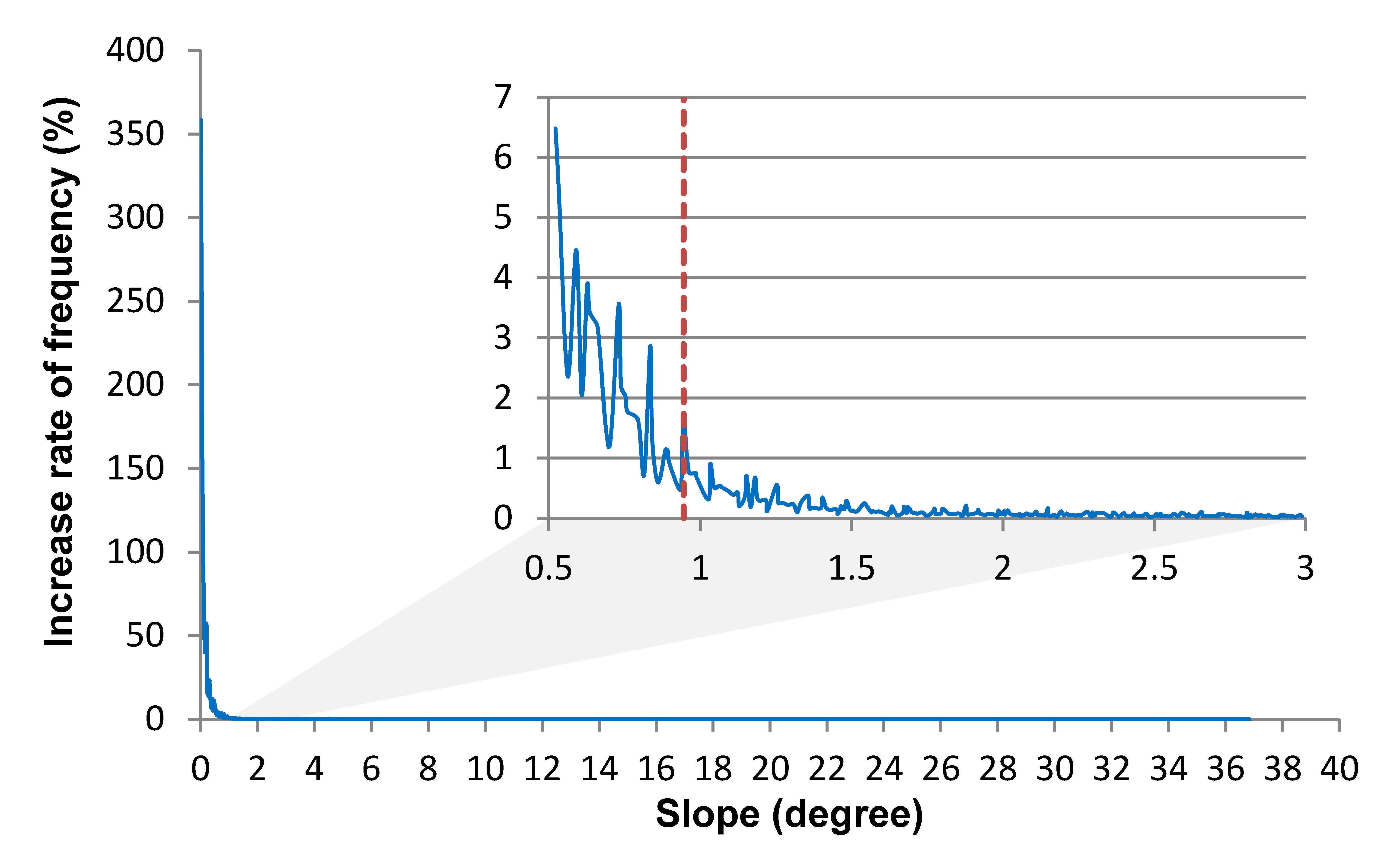

Supplement: Supplementary file 1 — Additional file 1: Figure S1. The increase rate of frequency by slope from low to high. The increase rate of frequency was defined as the frequency at a specific slope was divided by the accumulated frequency at all observed slopes that were less than the specific slope. [file 13071_2022_5496_MOESM1_ESM.jpg]

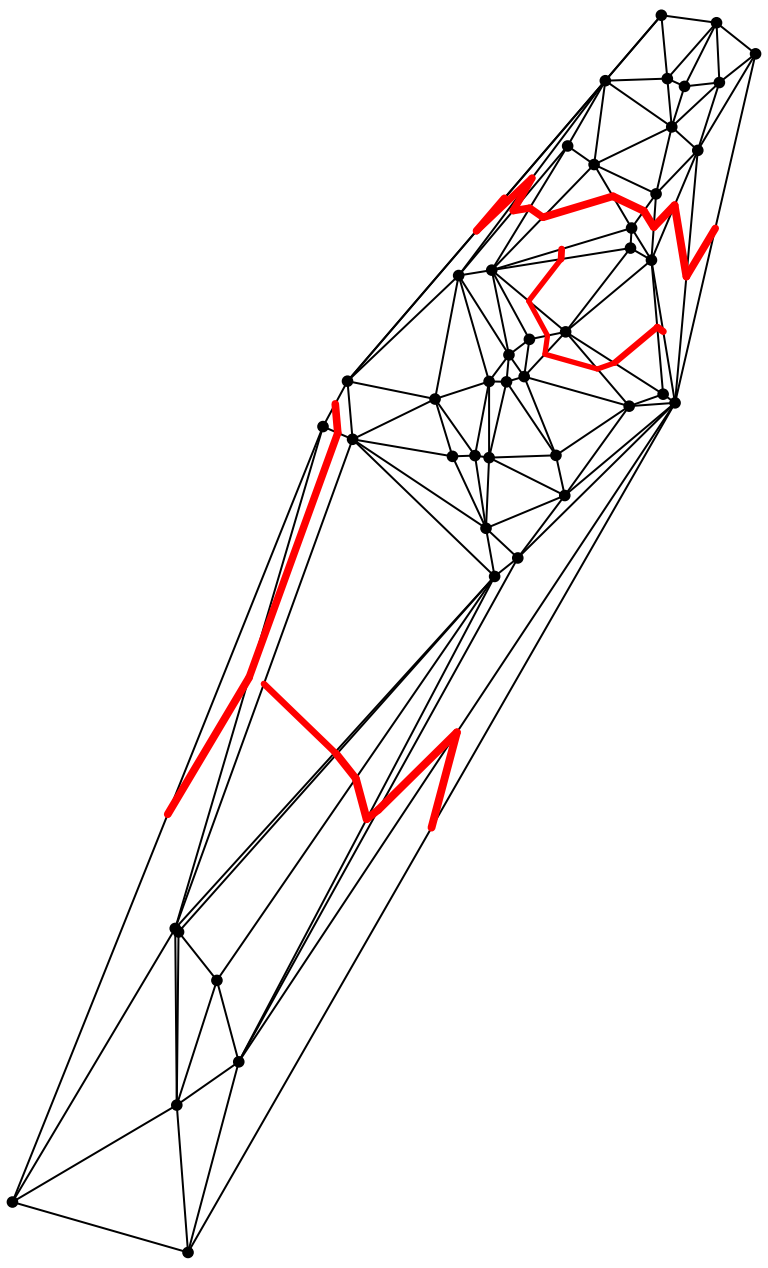

Supplement: Supplementary file 3 — Additional file 3: Figure S2. Genetic barriers for the snail populations based on Monmonier’s algorithm. [file 13071_2022_5496_MOESM3_ESM.pdf]

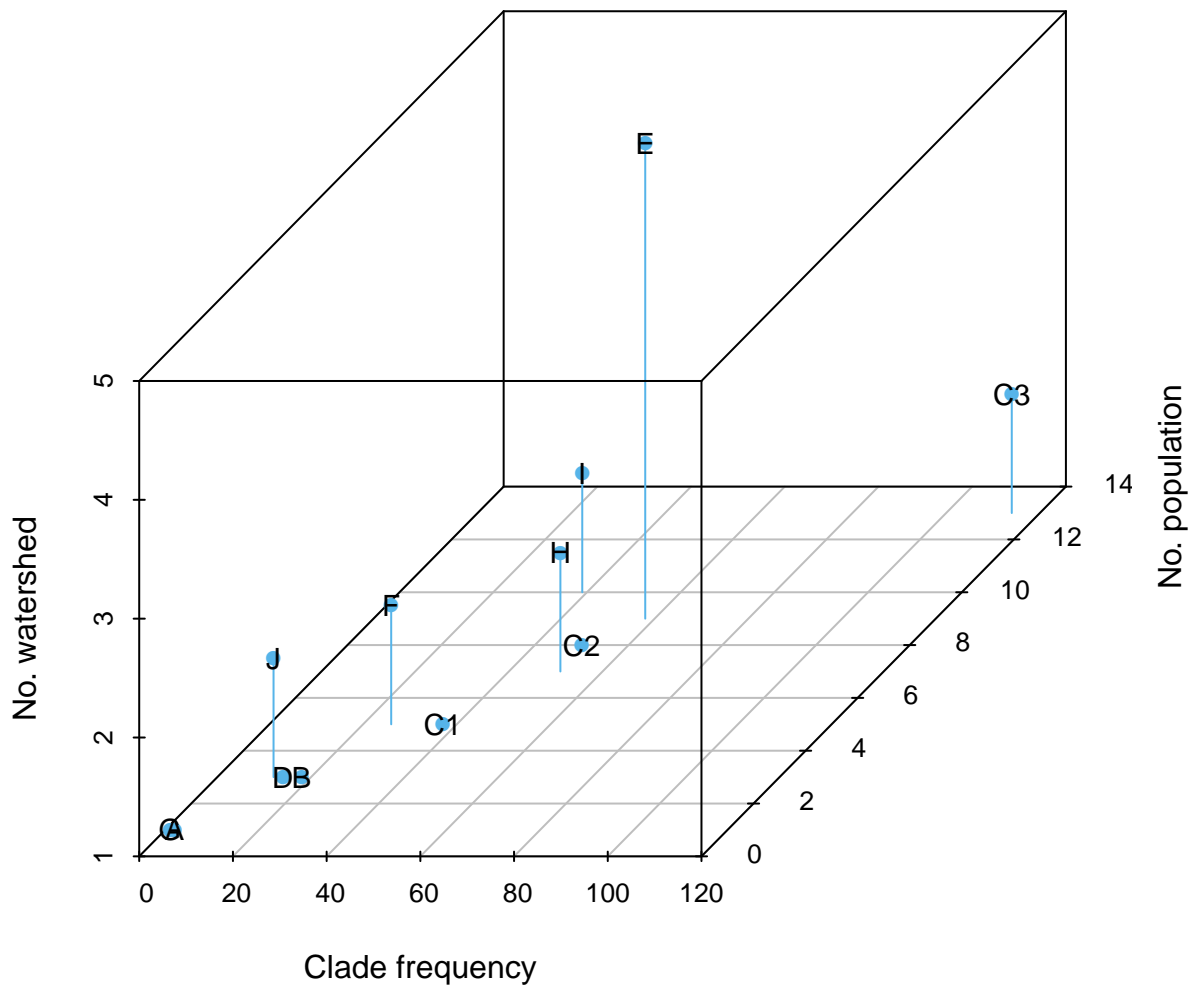

Supplement: Supplementary file 4 — Additional file 4: Figure S3. Three-dimensional distribution pattern of clades in populations, watersheds and frequency. [file 13071_2022_5496_MOESM4_ESM.pdf]
