# Supplementary material for: Cross-watershed distribution pattern challenging the elimination of Oncomelania hupensis, the intermediate host of Schistosoma japonica, in Sichuan province, China
Source: Parasit Vectors. 2022 Oct 11;15:363. doi: 10.1186/s13071-022-05496-0 (PMC9555091; doi:10.1186/s13071-022-05496-0)
Supplement: Supplementary file 5 — Additional file 5: Figure S4. A Kruskal-Wallis comparison analysis of median distances among different groups. [file 13071_2022_5496_MOESM5_ESM.pdf]

Pairwise Comparisons of VAR00002

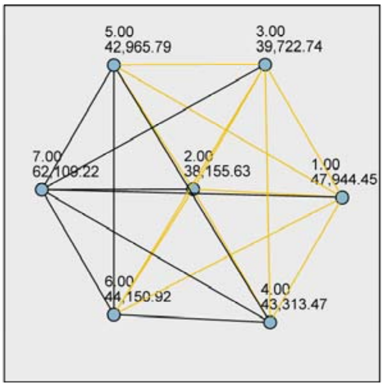

Each node shows the sample average rank…

Sample1-Sample2	Test Statistic	Std. Error	Std. Test Statistic	Sig.	Adj.Sig.
2.00-3.00	-1,567.111	299.369	-5.235	.000	.000
2.00-5.00	-4,810.162	469.296	-10.250	.000	.000
2.00-4.00	-5,157.843	421.465	-12.238	.000	.000
2.00-6.00	-5,995.290	900.657	-6.657	.000	.000
2.00-1.00	9,788.824	228.924	42.760	.000	.000
2.00-7.00	-23,953.594	8,545.962	-2.803	.005	.106
3.00-5.00	-3,243.051	481.080	-6.741	.000	.000
3.00-4.00	-3,590.732	434.548	-8.263	.000	.000
3.00-6.00	-4,428.180	906.853	-4.883	.000	.000
3.00-1.00	8,221.714	252.200	32.600	.000	.000
3.00-7.00	-22,386.484	8,546.617	-2.619	.009	.185
5.00-4.00	347.681	565.198	.615	.538	1.000
5.00-6.00	-1,185.128	976.217	-1.214	.225	1.000
5.00-1.00	4,978.662	440.706	11.297	.000	.000
5.00-7.00	-19,143.432	8,554.255	-2.238	.025	.530
4.00-6.00	-837.448	954.145	-.878	.380	1.000
4.00-1.00	4,630.982	389.379	11.893	.000	.000
4.00-7.00	-18,795.752	8,551.764	-2.198	.028	.587
6.00-1.00	3,793.534	886.096	4.281	.000	.000
6.00-7.00	-17,958.304	8,588.727	-2.091	.037	.767
1.00-7.00	-14,164.770	8,544.440	-1.658	.097	1.000

Each row tests the null hypothesis that the Sample 1 and Sample 2 distributions are the same.
Asymptotic significances (2-sided tests) are displayed. The significance level is .05.
